# Liquid biopsy in cancer diagnosis and prognosis: a paradigm shift in precision oncology

**DOI:** 10.3389/fmolb.2025.1708518

**Published:** 2026-01-12

**Authors:** Rayane da Silva Abreu, Danielle Dias Pinto Ferreira, Natassia Silva de Araujo, Samuel Horita, Tatiana Martins Tilli, Wim Degrave, Aline dos Santos Moreira, Mariana Caldas Waghabi

**Affiliations:** 1 Laboratory of Applied Genomics and Bioinovations, IOC, Fiocruz, Rio de Janeiro, Brazil; 2 Integrated Center for Translational Oncology Research (CIPOT), Rio de Janeiro, Brazil; 3 Laboratory of Clinical and Experimental Pathophysiology, IOC, Fiocruz, Rio de Janeiro, Brazil; 4 Translational Oncology Platform, Center for Technological Development in Health, Fiocruz, Rio de Janeiro, Brazil

**Keywords:** cancer diagnostics, cancer, circulating tumor DNA (ctDNA), circulating tumor cells (CTC), liquid biopsy, precision oncology

## Abstract

Liquid biopsy has emerged as a transformative tool in precision oncology, offering a minimally invasive approach for cancer detection, monitoring, and treatment guidance. Unlike traditional tissue biopsies, which are invasive and limited by tumor accessibility and sampling bias, liquid biopsy enables real-time tumor assessment through the analysis of circulating biomarkers in blood and other biofluids. This review provides a comprehensive overview of recent advances in liquid biopsy, with a focus on circulating tumor cells (CTCs), circulating tumor DNA (ctDNA), non-coding RNAs, extracellular vesicles (exosomes), and secreted proteins. These biomarkers offer valuable insights into tumor biology, supporting applications in early diagnosis, prognosis, treatment response monitoring, and minimal residual disease detection across various cancer types. We also discuss state-of-the-art methodologies, including next-generation sequencing, digital PCR, microfluidics, proteomics, and emerging artificial intelligence–based approaches that enhance the sensitivity, specificity, and scalability of liquid biopsy assays. Clinical studies demonstrate the potential of liquid biopsy for tailoring targeted therapies, predicting resistance mechanisms, and identifying tumor recurrence earlier than conventional methods. Furthermore, FDA-approved assays and ongoing phase III and IV clinical trials highlight its growing integration into routine clinical practice. Beyond technical innovations, this review examines the global landscape of liquid biopsy, emphasizing opportunities and challenges for implementation across diverse healthcare settings. Disparities in access, particularly between high-income and low- and middle-income countries, underscore the need for strategies that ensure equitable adoption of liquid biopsy technologies worldwide. In summary, liquid biopsy represents a paradigm shift in oncology, bridging innovations in cancer diagnostics with clinical applications. By enabling dynamic, personalized, and less invasive cancer management, it holds great promise for improving patient outcomes and advancing precision medicine.

## 1 Introduction

The cancer diagnostic process is complex and often involves multiple stages, starting with the measurement of tumor markers together with imaging tests to assess the presence of tumor masses. Additionally, exploratory surgeries and invasive procedures are sometimes necessary to obtain tissue samples for further analysis. While tissue biopsy remains the gold standard for cancer diagnosis, it has significant limitations. The process of obtaining and analyzing tissue samples is not only time-consuming and costly but also carries inherent risks, such as complications from the procedure itself.

Moreover, biopsies may not always be feasible, particularly in cases of tumors that are difficult to access or in patients who are in fragile health. These factors can delay final diagnosis, reduce the effectiveness of early intervention, and ultimately negatively affect the patient’s clinical outcome.

Furthermore, the stage at which the cancer is diagnosed plays a critical role in treatment options and prognosis, with early detection significantly improving survival rates. Therefore, the conventional diagnostic approach remains challenging, particularly in resource-limited settings or when early-stage cancers are present without clear symptoms. Given these challenges, there is an increasing need for the development of novel screening methods, ideally non-invasive or minimally invasive, that can detect cancer at earlier stages. Such advancements could provide significant improvements in both early detection and patient outcomes, while minimizing the risks and costs associated with traditional diagnostic techniques.

## 2 Liquid biopsy

**FIGURE 1:**
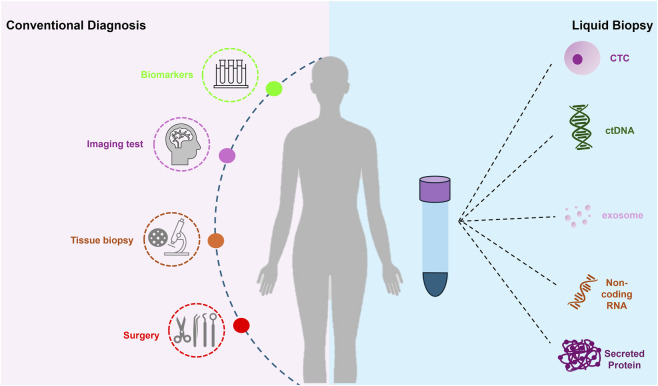
Cancer Diagnostic Process: stages of conventional cancer diagnosis compared to liquid biopsy methodology.

The well-established knowledge that tumors release cancer cells, nucleic acids, proteins, and extracellular vesicles into peripheral blood has enabled the development of more efficient cancer diagnostic methods, such as liquid biopsy. In the search for diagnostic approaches that streamline the cancer detection process, liquid biopsy has emerged as a simple, rapid, cost-effective, and minimally invasive alternative. Although initially focused on CTC detection, liquid biopsy has since evolved to include the identification of other tumor-derived components, such as circulating tumor DNA (ctDNA), cell-free circulating RNA (including non-coding RNA, mRNA, and miRNA), extracellular vesicles (exosomes) and secreted proteins (Figure 1) (Speicher and Pantel, 2014; Edsjö et al., 2023).

While blood is the minimally invasive primary source for biomarker detection in liquid biopsy, alternative fluids such as urine, saliva, breast milk, and ascitic fluid also show promise for sample collection (Jain et al., 2019; Satyal et al., 2019; Zhou et al., 2021; Nonaka and Wong, 2022; Saura et al., 2023).

While the scope of detectable tumor fragments *via* liquid biopsy continues to expand, most clinical studies have primarily focused on CTCs and ctDNA (Neumann et al., 2018; Pantel, 2021). The analysis of these biomarkers *via* liquid biopsy provides valuable insights into primary cancer detection, the molecular characterization of minimal residual disease, and prognostic assessments for patient survival across various solid tumor types (Pantel, 2021). Liquid biopsy has also been successfully applied to the early detection of lung and breast cancer (Ilie et al., 2014; Thery et al., 2019), colorectal and prostate cancer (Ligthart et al., 2013), evaluation of survival and response to neoadjuvant treatment in breast cancer patients (Magbanua et al., 2021), as well as monitoring and assessing local and distant recurrences (Bidard et al., 2008). In Table 1, the main biomarkers used in liquid biopsy are listed and the different detection methods are compared. Thus, liquid biopsy is increasingly integrated into the framework of personalized medicine, where mutational analyses enable the identification of targeted therapies directed to patient-specific molecular profiles.

**TABLE 1:** Biomarkers in liquid biopsy: an overview of the different biomarkers detected through liquid biopsy—CTCs, ctDNA, miRNAs, exosomes and secreted protein—their sources, and clinical applications.

Biomarker	Sources	Advantages	Disadvantages	Clinical applications
CTCs	Blood	Provide genomic and transcriptomic information	Low blood concentration	Monitoring disease progression
		Progression and survival indicators	Challenges in capture and analysis	Assessment of response to treatment
ctDNA	Blood	Early detection of cancer	Low concentration in cfDNA	Monitoring response to treatment
			Potential for false negative results	Detection of minimal residual disease (MRD)
Non-coding RNA	Blood	Stability in biofluids	Challenges in standardization and validation	Early detection of cancer
		Potential as biomarkers for various diseases	Bioavailability and systemic distribution	Monitoring disease progression
Exosomes	Blood, urine, saliva	Contain genetic material and proteins	Complexity in purification and analysis	Diagnosis and monitoring of various diseases
		May reflect tumor status and response to treatment	Need for advanced techniques for analysis	Identification of new therapeutic targets
Secreted proteins	Blood, urine, saliva	Post-translational informations	Expression variability	Diagnosis and monitoring of various diseases
		Proteins directly carry out cellular functions and primary targets in most current cancer therapies	Influence of conditions unrelated to cancer	Identification of new therapeutic targets

### 2.1 Exploring the different approaches to liquid biopsy

#### 2.1.1 Circulating tumor cells (CTCs)

CTCs are rare tumor-derived cells that circulate in the bloodstream at very low concentrations. Their estimated frequency ranges from 1 to 10 CTCs per 10^6^ to 10^8^ white blood cells (Neumann et al., 2018). This rarity makes their detection, isolation, and quantification particularly challenging, requiring highly sensitive methodologies.

The development of a sensitive, efficient, and reliable method for isolating CTCs remains the main challenge for the widespread application of this technique. Various approaches have been described to separate CTCs from normal blood cells. These methods can be based on physical properties, such as cell size and density—CTCs range from 4 to 50 µm (Stoecklein et al., 2016) and are more rigid than hematopoietic cells (Bagnall et al., 2015)—or on biological properties, such as the expression of specific surface markers (Neumann et al., 2018).

**FIGURE 2:**
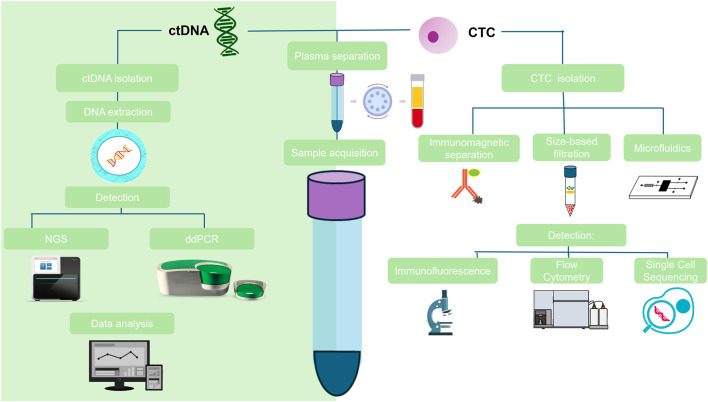
Detection of ctDNA vs. CTCs: stages of the process for detecting circulating tumor DNA (ctDNA) and circulating tumor cells (CTCs) from blood sample collection and analysis.

Current methods for CTC separation operate in two stages (Speicher and Pantel, 2014): primary enrichment and (Edsjö et al., 2023) detection and counting of CTCs (Figure 2). Primary enrichment can be achieved through positive or negative immunoselection using membrane antigens such as MUC1, EpCAM, and CD45, or through filtration methods based on CTC size. Following enrichment, CTC detection and counting are performed using techniques that enable optical visualization of the cells, such as nuclear staining and epithelial antigen labeling (Thery et al., 2019).

The presence of CTCs in the bloodstream is closely linked to the onset of metastasis, a process in which cancer cells detach from the primary tumor, enter the bloodstream, and migrate to distant sites (Thery et al., 2019). Although thousands of malignant cells from primary tumors are released into circulation, only a small fraction (approximately 0.1%) survive and successfully establish distant metastases (Wirtz et al., 2011). Studies have shown that CTC detection correlates with a worse prognosis and decreased progression-free and overall survival in metastatic breast and prostate cancer (Cristofanilli et al., 2004; Bono et al., 2008).

Given the potential of circulating tumor cells (CTCs) as tumor biomarkers, there is increasing interest in developing highly sensitive and effective methods for their enrichment and detection. Simple approaches based on cell density, such as gradient centrifugation, provide a straightforward means of isolating specific cell types. However, these methods often suffer from contamination by peripheral blood cells, which can limit their overall effectiveness. To overcome this challenge, the RareCyte system utilizes cell density properties while integrating sample preparation, imaging, and single-cell recovery steps, enhancing the precision and reliability of CTC analysis (Ramirez et al., 2017).

Size-based filtration methods are also being explored for CTC isolation. One such approach is ISET (Isolation by Size of Epithelial Tumor Cells), which enables the counting and characterization of CTCs from peripheral blood samples. This technique has been successfully applied to patients with hepatocellular carcinoma undergoing tumor resection (Vona et al., 2000). Another system, VyCap, is an immunoassay platform utilizing 5 μm microsieves, specifically designed for isolating CTCs in lung cancer cases (Lampignano et al., 2015; Tamminga et al., 2020). Additionally, OncoQuick combines both size- and density-based principles for CTC separation, employing a column with a porous membrane in the middle for effective filtration (Ferreira et al., 2016).

Microfluidic systems are also being employed for CTC enrichment, offering an efficient means of isolating these cells from other blood components through continuous flow. One such platform, Parsortix®, utilizes a size-based microfluidic approach to recover CTCs from processed blood samples. It features a filtration cartridge with microchannels ranging from 6.5 to 10 μm in width, where CTCs (>8 μm) are selectively trapped and subsequently stained with immunofluorescent antibodies (Kitz et al., 2021). In addition to Parsortix®, other microfluidic-based systems, such as ClearCell® FX1 (Khoo et al., 2014) and Vortex (Sollier et al., 2014), have also been developed for efficient CTC isolation.

Although numerous methods for CTC enrichment and separation have been developed, only one has received FDA approval to date: CellSearch®. This system utilizes an immunomagnetic approach based on EpCAM expression on the surface of CTCs. CTC detection is performed through the analysis of three key markers: CD45, for negative selection to eliminate leukocytes and EpCAM and cytokeratin, for positive selection (Neumann et al., 2018). The CellSearch® assay has received FDA approval for clinical use in monitoring patients with metastatic breast, prostate, and colorectal cancers (Swennenhuis et al., 2016).

The CellSearch® assay was employed to evaluate the clinical validity of circulating tumor cell (CTC) quantification as a prognostic tool for patients with metastatic breast cancer. A pooled analysis comprising 1,944 patients from 20 studies across 17 centers assessed the prognostic significance of CTC enumeration. At baseline, 46.9% of patients presented with ≥5 CTCs per 7.5 mL of blood, a threshold that was significantly associated with reduced progression-free survival (HR = 1.92; *p* < 0.0001) and overall survival (HR = 2.78; *p* < 0.0001). Elevated CTC counts at 3–5 and 6–8 weeks following treatment initiation were also correlated with poorer survival outcomes. Incorporating baseline CTC levels into clinicopathological models significantly enhanced survival prediction, which was further improved by tracking CTC dynamics throughout therapy (Bidard et al., 2014).

Additionally, the prognostic value of changes in CTC counts was also evaluated using the CellSearch® system in patients with metastatic breast and prostate cancers undergoing chemotherapy. CTC enumeration was conducted in 111 breast cancer patients and 185 prostate cancer patients, both before and after therapy initiation. The study concluded that the primary therapeutic goal should be the complete elimination of CTCs, a response that may require 10–12 weeks of treatment. Failure to achieve a reduction in CTC levels within this timeframe indicates treatment inefficacy, supporting timely modifications in therapeutic strategy and reinforcing the utility of CTCs as dynamic biomarkers for monitoring treatment response (Coumans et al., 2012).

#### 2.1.2 Circulating tumor DNA (ctDNA)

Initially developed for CTC detection, liquid biopsy was soon expanded to include ctDNA analysis, establishing it as a promising tool for identifying oncogenic mutations. In cancer patients, ctDNA is present in peripheral blood, primarily released by tumor cells undergoing apoptosis or necrosis (Lin et al., 2021).

However, tumor cells are not the sole source of circulating DNA fragments. Non-tumor cells also release cell-free DNA (cfDNA), with ctDNA comprising only a small fraction of the total cfDNA. In early disease stages, ctDNA accounts for approximately 1% of circulating cfDNA, increasing to up to 40% in advanced stages (Bettegowda et al., 2014). Compared to cfDNA, ctDNA is typically more fragmented, with an average length of 150 bp, whereas cfDNA strands are generally longer (Mouliere et al., 2011). Since ctDNA carries mutations from the original tumor, its analysis enables the detection of tumor-specific genetic alterations, offering valuable insights into the molecular characteristics of the primary tumor. Advances in high-throughput sequencing and the Cancer Genome Project have significantly enhanced the utility of ctDNA in cancer detection (Cheng et al., 2016).

The clinical applications of ctDNA detection have been demonstrated in various studies, including (Speicher and Pantel, 2014): diagnosis and prognosis of non-small cell lung cancer (NSCLC), Hodgkin lymphoma, and hepatocellular carcinoma (García-Pardo et al., 2023; Alcoceba et al., 2021; Xu et al., 2017); (Edsjö et al., 2023) personalized therapy by guiding treatment decisions based on oncogenic mutation profiling in NSCLC (Uemura et al., 2023); (Jain et al., 2019) monitoring treatment response and therapy resistance in NSCLC, recurrent osteosarcoma, and nasopharyngeal cancer (Falk et al., 2023; Dhir et al., 2024; Lv et al., 2024); and (Satyal et al., 2019) early recurrence detection in colorectal and gastric cancer (Baraniskin et al., 2023; Hamakawa et al., 2015). Moreover, studies have shown that ctDNA levels progressively increase with disease progression (Bettegowda et al., 2014) and that ctDNA-based monitoring enables the detection of cancer recurrence an average of 10 months earlier than conventional methods (Reinert et al., 2016).

Due to its highly fragmented nature and low concentrations in blood samples, circulating tumor DNA (ctDNA) detection requires highly sensitive and specific techniques capable of enriching the analyzed material. Given the low abundance of these molecules in peripheral blood, high-throughput sequencing techniques marked the first major advancements in ctDNA detection (Cheng et al., 2016).

High-throughput sequencing methods have demonstrated promising results for ctDNA analysis (Figure 2). Next-generation sequencing (NGS)-based techniques offer high specificity by evaluating targeted gene panels and detecting point mutations, copy number variations, structural rearrangements, and methylation changes (Elazezy and Joosse, 2018). Tam-Seq, a deep amplicon sequencing method, enables the sequencing of large ctDNA regions and the detection of low-frequency tumor mutations. It successfully identified mutations with allele frequencies as low as 2% in plasma samples from high-grade serous ovarian cancer patients, achieving sensitivity and specificity above 97% (Forshew et al., 2012).

Another advanced technique, CAPP-Seq (Cancer Personalized Profiling by Deep Sequencing), combines optimized library preparation for low DNA input with a multi-step bioinformatics pipeline to design custom selector panels. These selectors use biotinylated oligonucleotides complementary to recurrent cancer-associated mutations, enabling highly sensitive ctDNA detection (Newman et al., 2014). In non-small cell lung cancer (NSCLC) patients, CAPP-Seq identified mutations in over 95% of tumors, detecting ctDNA in 100% of stage II-IV and 50% of stage I patients, with 96% specificity for mutant allele fractions as low as 0.02% (Newman et al., 2014).

Beyond sequencing approaches, digital PCR (dPCR)-based techniques are gaining attention for their ability to detect point mutations even at extremely low allele frequencies. Two widely used dPCR methods for ctDNA detection are Droplet Digital PCR (ddPCR) and BEAMing (beads, emulsion, amplification, and magnetism) (Elazezy and Joosse, 2018). ddPCR, a second-generation platform, employs water-oil emulsion droplet partitioning, with each droplet encapsulating a single DNA strand, either wild-type or mutant. This technique has successfully detected early-stage breast cancer in plasma by identifying PIK3CA mutations, achieving 93.3% sensitivity and 100% specificity (Beaver et al., 2014). While ddPCR offers high sensitivity and absolute quantification, it is limited to tracking known genetic variants.

In contrast, BEAMing (Beads, Emulsion, Amplification, and Magnetics) combines emulsion PCR with magnetic bead-based detection to amplify target DNA sequences using primers containing predefined tag sequences, which are subsequently bound to magnetic beads. These beads are then analyzed and classified *via* flow cytometry to detect mutation-positive events (Cheng et al., 2016). This technique has been successfully applied to detect activating and resistance mutations in the epidermal growth factor receptor (EGFR) gene from lung cancer ctDNA. In another study, a total of 44 patients were analyzed, including 23 patients with progressive disease following EGFR tyrosine kinase inhibitor (EGFR-TKI) therapy and 21 treatment-naïve individuals. Activating mutations were identified in 72.7% of plasma samples, while the T790M resistance mutation was detected in 43.5% of previously treated patients. The T790M allele fraction varied from 13.3% to 94%, with a peak mutation allele fraction ranging between 0.1% and 1%. These findings underscore BEAMing’s high sensitivity and specificity in quantifying T790M-positive alleles among those harboring activating mutations, offering a reliable tool for monitoring mutation dynamics and disease progression during targeted therapy (Taniguchi et al., 2011).

#### 2.1.3 Non-coding RNA (miRNA and lncRNA)

Advances in research on non-coding RNAs (ncRNAs) and their role in cancer pathogenesis have highlighted their involvement in key stages of disease development. Their expression levels are often dysregulated across various cancer types, reinforcing their potential as disease biomarkers, particularly in minimally invasive approaches such as liquid biopsy (Toden and Goel, 2022).

Most studies on ncRNAs in liquid biopsy focus on two key molecule types (Speicher and Pantel, 2014): microRNAs (miRNAs), small single-stranded RNAs (18–23 nt) that regulate gene expression post-transcriptionally, and (Edsjö et al., 2023) long non-coding RNAs (lncRNAs), transcripts longer than 200 nt that do not encode proteins but influence gene transcription and affect protein function and stability through molecular interactions (Ma et al., 2024).

Detection methods for ncRNAs in liquid biopsy are rapidly evolving, incorporating techniques such as qPCR, *in situ* hybridization, RNA microarrays, and RNA sequencing (Ma et al., 2024). One such approach involves circulating microRNAs (miRNAs), which have shown potential in melanoma diagnosis and risk stratification. A multicenter case-control study analyzed plasma miRNA profiles from 372 melanoma patients and 210 controls, leading to the identification of the MEL38 signature. This miRNA-based molecular signature demonstrated high diagnostic accuracy, with 93% sensitivity, 98% specificity, and an AUC of 0.98. Additionally, a novel prognostic panel, MEL12, stratified patients into low-, intermediate-, and high-risk groups, with 10-year survival rates of 94%, 78%, and 58%, respectively. MEL12 also correlated with clinical staging and sentinel lymph node biopsy status, revealing that high-risk patients were three times more likely to develop lymph node metastases. These findings highlight the potential of miRNA signatures to improve melanoma diagnosis and support personalized treatment strategies (Van Laar et al., 2023).

Another study explored serum-based m6A-modified miRNAs (m6A-miRNAs) for pan-cancer detection, utilizing a support vector machine (SVM) algorithm on a large cohort of 7,299 samples. The identified m6A-miRNA signature demonstrated exceptional accuracy, with AUCs of 0.979 (training set), 0.976 (internal validation), and 0.936 (external validation). This biomarker signature remained highly consistent across multiple cancer types, including lung, gastric, and hepatocellular carcinoma. These results suggest that m6A-miRNAs represent an accurate, accessible, and minimally invasive biomarker for population-based cancer screening (Zhang et al., 2021).

A recent study evaluated RNA from extracellular vesicles (EVs) as potential biomarkers for liquid biopsy, comparing different plasma collection methods. Researchers analyzed mRNAs (CK19, PCTK1), lncRNA (MALAT1), and miRNAs (miR21, miR155) in EVs isolated *via* differential centrifugation or annexin A5-coated magnetic beads (ANX beads). The results revealed that mRNAs and lncRNAs were predominantly enriched in larger EVs, while miRNAs were detected across multiple EV sizes. Notably, ANX bead capture significantly enhanced extracellular RNA (exRNA) detection in plasma, revealing higher levels of CK19, MALAT1, and miR155 in lung cancer patients compared to controls. These findings highlight the impact of plasma collection methods on exRNA analysis and suggest that ANX beads could serve as an effective tool for EV-based biomarker detection (Wang et al., 2022).

#### 2.1.4 Extracellular vesicles (exosomes)

Exosomes are extracellular vesicles secreted by various cell types and can be detected in multiple biological fluids, including blood, saliva, and urine. In cancer, they play a crucial role in regulating the tumor microenvironment and promoting tumor proliferation and progression (Zhou et al., 2021; Ma et al., 2024).

Compared to other liquid biopsy targets, such as CTCs and ctDNA, exosome detection offers several advantages (Speicher and Pantel, 2014): exosomes are more abundant in circulation than CTCs (Edsjö et al., 2023), they exhibit greater stability than ctDNA, which degrades rapidly, and (Jain et al., 2019) they can be detected in a wide range of bodily fluids, including blood, urine, saliva, ascites, bile, amniotic fluid, and cerebrospinal fluid (Zhou et al., 2021).

The promising potential of exosomes as biomarkers for liquid biopsy has driven the development of effective methods for their detection and isolation. Exosome analysis involves two main steps: enrichment followed by content analysis. For isolation and enrichment, commonly used techniques include differential ultracentrifugation, size-based separation, immunomagnetic separation, and microfluidics. Detection of exosomal content is performed using methods such as RT-PCR, genome sequencing, and proteomics (Ma et al., 2024).

A study assessed exosome-based detection as a biomarker for liquid biopsy, analyzing 581 plasma samples (431 from lung cancer patients and 150 controls). Researchers used an extracellular vesicle (EV) array with 49 antibodies to capture exosomes, followed by detection using biotin-conjugated CD9, CD81, and CD63 antibodies. Multimarker models were evaluated using AUC analysis and random forest algorithms. The most effective single markers for distinguishing cancer patients from controls were CD151 (AUC = 0.68), CD171 (AUC = 0.60), and TSPAN8 (AUC = 0.60). The top-performing model, combining 10 markers, achieved an AUC of 0.74 across all lung cancer subtypes and 0.76 for adenocarcinoma. In squamous cell carcinoma (SCC) and small cell lung cancer (SCLC), CD151 alone performed comparably to the multimarker model. These findings underscore the potential of exosome proteomics as a promising approach for lung cancer diagnosis, independent of cancer stage or histological subtype (Sandfeld-Paulsen et al., 2016).

#### 2.1.5 Secreted proteins

Proteomics is a powerful tool for identifying clinical biomarkers in liquid biopsies, offering real-time insights into cancer’s molecular dynamics. While DNA alterations indicate mutations and genetic predispositions, proteomic analysis reveals their functional impact on disease-related biological processes.

In recent decades, several protein biomarkers have been developed for clinical diagnostics, many of which have received FDA approval. Widely used examples include PSA (prostate cancer), CA 125 (ovarian cancer), and CA 19-9 (pancreatic cancer), which assist in diagnosis, treatment monitoring, and recurrence detection. However, early cancer detection using protein biomarkers remains challenging due to limitations in sensitivity and specificity. Traditional antibody-based methods, such as ELISA, IHC, and bead-based immunoassays, have been essential for protein detection but lack scalability for high-plex proteomic profiling. Since proteins are key effectors of cellular functions and primary drug targets in cancer therapy, high-dimensional proteomic analyses hold great promise for identifying novel biomarkers and improving clinical applications. Moreover, protein profiling in liquid biopsy samples may provide more organ-specific insights than DNA or RNA, aiding in tumor origin identification. Advances in proteomic technologies are expanding biomarker discovery, driving the development of innovative methods to overcome current limitations and enhance liquid biopsy applications (Ding et al., 2022).

Considering liquid biopsy approaches based on the detection of secreted proteins, a study investigating blood-based diagnostic tests for colorectal cancer (CRC) identified novel protein biomarkers with potential for early detection. Using mass spectrometry (SWATH-MS) combined with high-abundance protein ultradepletion and extensive peptide fractionation, 513 plasma proteins were identified from a cohort of 100 samples. Differential expression analysis revealed 37 biomarker candidates across CRC stages, with seven (CST3, GPX3, CFD, MRC1, COMP, PON1, ADAMDEC1) validated through Western blotting and ELISA. A neural network classification model further refined the panel to five proteins (SAA2, APCS, APOA4, F2, AMBP) capable of distinguishing early (I/II) from late-stage (III/IV) CRC. These findings highlight the potential of MS-based proteomics combined with ultradepletion strategies for developing highly compliant and effective blood-based CRC diagnostics (Ahn et al., 2019).

It is important to highlight that a protein biomarker panel-based test has been FDA-approved and successfully implemented in clinical practice for early-stage ovarian cancer detection. The Overa (MIA2G) test represents a significant advancement in ovarian cancer detection, building upon the foundation established by its predecessor, OVA1. Both assays utilize a multivariate index approach to assess malignancy risk in women with adnexal masses, but Overa incorporates an optimized biomarker panel—CA 125, apolipoprotein A-1, transferrin, follicle-stimulating hormone, and human epididymal protein 4—along with a refined algorithm to enhance diagnostic accuracy. While OVA1, approved in 2009, demonstrated high sensitivity, particularly for early-stage cancers, it also exhibited a high false-positive rate for benign masses. Overa, approved in 2016, was specifically designed to address this limitation by maintaining robust sensitivity while significantly improving specificity (Overa, 2025; FDA-Approved Tests, 2025).

A study evaluating the use of Overa as a reflex test for OVA1 demonstrated a significant improvement in the specificity of ovarian cancer detection in patients with adnexal masses. In this study, 1,035 serum samples were categorized into low, intermediate, and high cancer risk based on OVA1 scores, with intermediate-risk samples further analyzed using Overa. This approach reduced false positives by 58%, improving specificity from 50% to 72%, a result confirmed in an independent validation set (N = 207), where specificity increased from 56% to 73%. These findings highlight that integrating Overa as a reflex test for OVA1 significantly decreases false positives, minimizing unnecessary surgical referrals and enhancing the clinical utility of liquid biopsy in ovarian cancer assessment (Fritsche and Bullock, 2023).

### 2.2 Artificial intelligence in liquid biopsy

Bioinformatics has been instrumental in advancing liquid biopsy as a robust tool for cancer diagnostics and monitoring. By integrating high-throughput sequencing, multi-omic data analysis, and computational modeling, bioinformatics pipelines enable efficient processing and interpretation of complex datasets from cfDNA, CTCs, extracellular vesicles, and other analytes (Amorim et al., 2017; Manier et al., 2018; Tsui et al., 2021). These approaches identify tumor-specific alterations—such as somatic mutations, copy number variations (CNVs), and methylation patterns—supporting early detection, disease classification, and longitudinal monitoring (Gao et al., 2024; Yu et al., 2024; Crook et al., 2021; Widschwendter et al., 2017). Beyond biomarker discovery, bioinformatics contributes to assay optimization, quality control, and standardization of analytical workflows, thereby creating a computational foundation upon which artificial intelligence (AI) approaches can be built.

Machine learning (ML), a core component of AI, is propelling liquid biopsy toward greater sensitivity, specificity, and scalability. By leveraging deep architectures and advanced pattern-recognition algorithms, ML enables the extraction of complex biological signatures for applications ranging from early cancer detection and tumor subtyping to treatment monitoring and recurrence prediction. In the analysis of cellular and exosomal components, ML models—particularly convolutional neural networks (CNNs) and boosting methods—enhance the quantification and classification of CTCs and tumor-educated platelets (TEPs), improving disease monitoring and subtype discrimination (Poellmann et al., 2023; Pirone et al., 2023; Eledkawy et al., 2024). Similarly, multi-analyte models integrating cfDNA/ctDNA, protein markers, and CNVs have achieved high diagnostic accuracy across cancer types (Cygert et al., 2023; Kwon et al., 2023), while deep learning applied to circulating microRNA data enhances tissue-of-origin prediction (Raghu et al., 2024). Deep learning models can also predict cancer risk from cfDNA genomic signatures, even at low sequencing depths (Li et al., 2021).

These advances rely on a range of algorithmic frameworks that differ according to data type and analytical goal. Supervised learning methods—such as random forests, support vector machines (SVMs), and gradient boosting (XGBoost, LightGBM) — remain the standard for structured molecular data, while deep learning architectures, including CNNs and transformers, are increasingly applied to sequencing, imaging, and spectroscopic data (Eledkawy et al., 2024; Halicek et al., 2017; Wang et al., 2025; Lee et al., 2023). Ensemble strategies that integrate these complementary models can further enhance generalizability and robustness across heterogeneous datasets (Pasha Syed et al., 2022). To ensure reliability, models must undergo rigorous validation—for instance employing k-fold or nested cross-validation and independent cohort testing to prevent overfitting (Gu and Lu, 2025; Clift et al., 2023; Xu et al., 2022). Performance is then assessed using AUC-ROC, sensitivity, specificity, precision–recall metrics, and F1 score, while calibration and decision curve analyses provide additional insight into interpretability and clinical utility (Gu and Lu, 2025; Klontzas et al., 2025).

Beyond genomics, machine learning has been increasingly applied to non-genomic analytes and alternative biofluids within liquid biopsy workflows. In metabolomics, ML applied to serum LC-ESI-MS profiles has enabled highly sensitive early detection of pancreatic cancer (Iwano et al., 2021). Spectroscopic approaches, such as Raman and infrared spectroscopy, similarly benefit from ML-based pattern recognition, facilitating molecular fingerprinting and cancer subtype classification from plasma exosomes (Tipatet et al., 2025; Oktem et al., 2025; Kim et al., 2023). Extending beyond blood-based assays, ML analysis of urinary exosomal microRNA signatures integrated with clinical variables has improved bladder cancer diagnosis (Bitiņa-Barlote et al., 2025).

In summary, AI is transforming liquid biopsy into a sensitive, scalable, and clinically actionable platform for early cancer detection and monitoring. As algorithms and datasets mature, progress in model interpretability, standardization, and clinical validation will be essential to achieve broad clinical adoption and patient benefit.

### 2.3 Liquid biopsy for the detection of minimal residual disease

Minimal Residual Disease (MRD) refers to the presence of microscopic levels of cancer cells that persist after initial treatment and are below the detection threshold of conventional imaging and clinical markers. Liquid biopsy has emerged as a valuable tool for MRD detection due to its capacity to identify minimal amounts of tumor-derived molecules, as CTC and ctDNA, in plasma with high sensitivity (Honoré et al., 2021).

Liquid biopsy–based MRD detection relies on two main strategies: tissue-informed and liquid-only approaches. They differ primarily in the need for prior tumor sequencing. Tissue-informed methods use tumor-specific genomic profiling to create personalized assays that monitor patient-specific mutations, while liquid-only methods assess circulating biomarkers directly in plasma using predefined genomic or epigenomic panels. This distinction shapes key performance and practical aspects of MRD testing, including sensitivity, specificity, clinical applicability, turnaround time, and overall cost (Boukouris et al., 2025).

Several commercial assays exemplify the clinical implementation of liquid biopsy for MRD detection, employing either tissue-informed or liquid-only strategies. Among the tissue-informed assays, Signatera™ is a leading example, generating personalized panels based on tumor-specific mutations to enable highly specific longitudinal monitoring. In contrast, liquid-only platforms rely on predefined genomic or epigenomic signatures detectable directly in plasma. Guardant Reveal™ exemplifies this approach by integrating genomic and methylation-based signals for MRD detection and recurrence monitoring without requiring tumor tissue. Other widely used liquid-only assays include FoundationOne Liquid CDx™, which provides broad genomic profiling for multiple solid tumors, and GRAIL Galleri®, which applies methylation-based classification to detect circulating tumor signals and support early cancer detection. Together, these platforms illustrate the diversity of technological strategies currently applied to MRD detection (Boukouris et al., 2025).

### 2.4 Liquid biopsy and clinical trials: Future perspectives

Liquid biopsy has emerged as a promising tool in oncology, according to ClinicalTrials.gov, numerous ongoing clinical trials are currently investigating its application in various clinical settings. Excluding “Terminated” or “Suspended” studies, 555 active trials are currently exploring its use, ranging from early cancer detection to monitoring treatment response and identifying resistance-associated mutations.

Among these, seven completed trials stand out as the most advanced and closest to clinical implementation (Table 2). Their findings will be crucial in shaping the future of liquid biopsy in routine practice.

**TABLE 2:** Clinical studies: summary of completed clinical trials that have used liquid biopsy for cancer diagnosis, monitoring or treatment.

Type of cancer	Efficacy	Biomarker	Study phase	Clinical trial ID
Metastatic lung cancer and metastatic gastrointestinal cancer	To evaluate the impact of early liquid biopsy alongside standard diagnostics	ctDNA (FoundationOne® liquid CDx)	Phase IV	NCT05846594
NeuroEndocrine tumours (NET)	Predict clinical symptomatic response and progression-free survival in patients treated with Somatuline® Autogel®	CTC	Phase IV	NCT02075606
Metastatic breast cancer	To predict disease progression and response to treatment	Secreted protein and ctDNA	Phase III	NCT03439046
Colorectal cancer	The significant utility of tumor-expressed notch-associated lncRNAs as prognostic malignancy indicators in patients with CRC	Long non-coding RNA	Not applicable	NCT06432413
Lung cancer	The molecular signature of the 26 genes (LungCancerTest) revealed in blood and respiratory fluids	RNA	Not applicable	NCT02853006
Lymphoma	Develop liquid biopsy technology for accurate diagnosis and prognosis judgment of lymphoma	cfDNA	Not applicable	NCT04062877
Prostate cancer	Establishment of a diagnostic, prognostic and active surveillance test for prostate cancer	Long non-coding RNA	Not applicable	NCT05141383

FoundationOne® Liquid CDx, was FDA-approved liquid biopsy for clinical and commercial use. By analyzing circulating tumor DNA (ctDNA) from a blood sample, the test provides comprehensive genomic profiling, evaluating over genes, offering a faster, less invasive alternative to tissue biopsy. This prospective international Phase IV study (NCT05846594) enrolled participants with clinically suspected advanced metastatic cancer (lung or gastrointestinal), who had not yet received treatment for metastatic disease and had no confirmed pathological diagnosis. The trial aimed to determine whether incorporating this liquid biopsy into the standard diagnostic workflow could shorten the time to complete diagnosis and treatment initiation. Full outcome data on survival or treatment response are not yet available.

The pursuit of new technologies applied to liquid biopsy demonstrates the growing interest in more sensitive, minimally invasive methods capable of capturing tumor heterogeneity in real time. In this context, the clinical trials NCT02853006, NCT04062877, and NCT05141383 (Table 2) are aligned with this purpose, exploring different biomarkers and cancer types to validate innovative approaches. The NCT02853006 trial proposes an approach based on a 26-gene molecular signature detected in blood and respiratory fluid samples for lung cancer, aiming to improve diagnostic accuracy and therapeutic response monitoring. The NCT04062877 study investigates the application of liquid biopsy in lymphoma, seeking to enhance molecular detection and disease monitoring through circulating biomarkers, although efficacy results have not yet been disclosed. Meanwhile, NCT05141383 focuses on prostate cancer, assessing the diagnostic and prognostic potential of lncRNAs detected in blood and urine as a non-invasive alternative to traditional tissue biopsies. Collectively, these studies represent a strategic movement toward the personalization of oncology, pursuing less invasive, more sensitive, and dynamic methods for the clinical management of solid and hematologic tumors. Although efficacy results are not yet fully available, the potential impact of these approaches lies in their ability to enable early detection, real-time therapeutic monitoring, and reduction of invasive biopsy procedures, thereby consolidating liquid biopsy as an emerging pillar of precision medicine.

The data suggests that liquid biopsy is undergoing a transition from an experimental tool to a widely adopted clinical approach. However, challenges remain, particularly in standardizing methodologies and achieving large-scale clinical validation to ensure its full potential.

### 2.5 Global market for liquid biopsy products

As the global prevalence of cancer continues to rise, the need for early and precise diagnostic methods to optimize therapeutic management has become more critical than ever. As a result, the adoption of liquid biopsy, as a non-invasive approach, has been accelerating in recent years. The liquid biopsy market is experiencing rapid growth, with a diverse range of products and applications emerging to meet the growing demand for more accurate and minimally invasive diagnostic solutions.

As an example, the global market for liquid biopsy of CTCs has been growing in recent years. According to a study published by Fortune Business Insights, the global liquid biopsy market was valued at USD 8,01 billion in 2023, and is projected to reach USD 9,63 billion in 2024, with an anticipated growth to USD 58,64 billion by 2032, reflecting a compound annual growth rate (CAGR) exceeding 25% (https://www.fortunebusinessinsights.com/liquid-biopsy-market-102506). With this rapidly growing and expansive market, many industries are focusing on expanding their products within the liquid biopsy market. These include:

Blood Collection Tubes: Designed for the safe and efficient collection of blood samples, these tubes are crucial for maintaining the integrity of the sample until the completion of the assay, preventing degradation and ensuring accurate results.

Kits and Reagents: Essential for the collection and analysis of samples, these products play a critical role in preserving biomarkers during the assay, ensuring the reliability and accuracy of the results in liquid biopsy tests.

Devices or Systems: Specialized equipment designed to facilitate the detection and analysis of biomarkers in body fluids. The accuracy and sensitivity of these devices are critical for ensuring precise biomarker detection, which directly impacts the reliability and effectiveness of liquid biopsy assays.

Parallel technologies: Advancements in the emergence of new technologies, such as nanotechnology, NGS and digital PCR are driving the accuracy and sensitivity of liquid biopsy assays, expanding and improving their clinical and commercial applications.

A variety of liquid biopsy tests are currently available on the market, offering non-invasive options for cancer detection, monitoring, and treatment guidance. These tests utilize different methodologies, target various cancer types, and are offered by several companies. Table 3 summarizes some of the commercially available liquid biopsy products.

**TABLE 3:** Commercialized methods: list of liquid biopsy-based techniques that have been approved and are currently available on the market.

Product	Method type	Cost (*)	Target cancer type	Commercial provider
Guardant360® CDx	Genomic profiling test analyzing 55 genes *via* ctDNA, aiding in treatment selection for advanced-stage cancer patients	High cost	Multiple solid tumors	Guardant Health
Galleri®	A multi-cancer early detection test that analyzes cfDNA methylation patterns	Moderate cost	Pan-cancer (early detection)	GRAIL, Inc
LiquidHALLMARK®	Amplicon-based NGS (ctDNA + ctRNA): argets mutations in 80 genes and fusions in 10 genes, with an optional analysis of 10 ctRNA targets, providing insights for 15 cancer types	Moderate cost	Lung, breast, colorectal, others	Lucence
HelioLiver™	Combines cfDNA methylation patterns with serum protein markers	Moderate cost	Hepatocellular carcinoma	Helio Health/Fulgent Genetics
DefineMBC™	Integrates circulating tumor cell analysis with cfDNA sequencing	High cost	Metastatic breast cancer	Epic Sciences
FoundationOne® liquid CDx	NGS-based cfDNA comprehensive genomic profiling	High cost	Lung, breast, prostate and ovarian cancer	Foundation Medicine
OncoBEAM™ RAS CRC	Utilizes BEAMing technology to detect RAS mutations	Moderate cost	Colorectal cancer	Sysmex Inostics
SelectMDx®	A urine-based test detecting RNA biomarkers associated with prostate cancer, helping to reduce unnecessary biopsies	Low cost	Prostate cancer	MDxHealth
CxBladder®	A suite of urine-based tests using RNA and protein assays for the detection and monitoring of bladder cancer	Low cost	Bladder cancer	Pacific Edge Limited
CellSearch®	Immunomagnetic method for detection and counting of CTCs in blood	High cost	Metastatic breast, colorectal and prostate cancer	Menarini Silicon Biosystems
Signatera™ (MRD)	Tumor-informed NGS ctDNA test (patient-specific panel)	High cost	Multiple solid tumors	Natera
Guardant Reveal™ (MRD)	Tumor-agnostic ctDNA methylation + genomic alterations	High cost	Multiple solid tumors	Guardant Health
RaDaR™ (MRD)	Tumor-informed ultra-deep NGS ctDNA test	High cost	Breast, lung, colorectal, head and neck, and other solid tumors	Inivata/NeoGenomics

(*) Low cost = estimated cost below US$500 (screening or detection of specific markers using accessible technologies); Moderate cost = estimated cost between US$500 and US$2,000 (cancer diagnostics with a limited scope, such as targeting a few genes or a specific tumor type); High cost = estimated cost above US$2,000 (multi-gene sequencing, personalized analysis, or combined multi-omic profiling).

### 2.6 Integrating liquid biopsy into the oncology patient journey: opportunities and challenges

The use of liquid biopsy in cancer care offers both promising opportunities and notable challenges. One of the key advantages of this technique is its ability to provide a simple, fast, and minimally invasive method for diagnosing cancer early and monitoring treatment response, which can help improve clinical outcomes and personalize therapies for individual patients. By analyzing components such as CTCs and ctDNA, liquid biopsy can reveal important details about the tumor’s molecular profile and the presence of specific mutations, supporting more personalized treatment decisions.

However, there are challenges to address, such as the rarity of CTCs, which complicates their detection and quantification, and the need for highly sensitive methods to analyze ctDNA, which is often present in very low concentrations. Variability in sample quality and the interpretation of results can also make it difficult to implement liquid biopsy in clinical practice. Overall, while liquid biopsy represents a major advancement in cancer care, successfully integrating it into routine clinical practice will require overcoming technical barriers and continually validating its effectiveness in different oncological settings.

Liquid biopsy has the potential to transform the care of oncology patients, particularly when compared to traditional diagnostic methods. From early detection to monitoring treatment response, it offers a non-invasive way to identify CTCs and fragments of ctDNA in blood samples. This is especially valuable in the early stages of the disease, where early detection can significantly improve survival rates.

As a global health challenge, there are significant disparities in cancer diagnosis between high-income and low- and middle-income countries, particularly regarding access to advanced technologies such as liquid biopsy. In high-income countries, this method has already been integrated into routine clinical practice for treatment monitoring and detection of tumor mutations—especially in lung and colorectal cancers—supported by strong laboratory infrastructure, established regulatory frameworks, and greater financial resources (Siravegna et al., 2017; Rolfo et al., 2021). The adoption of liquid biopsy accelerated after the U.S. Food and Drug Administration (FDA) approved the first test in 2016 to detect EGFR mutations in non-small cell lung cancer. Since then, its use has expanded across multiple cancer types, including applications in early detection, treatment response monitoring, drug resistance assessment, and recurrence prediction (Pandey and Yadav, 2024). However, in Latin America, the adoption of this and other innovative cancer technologies faces major obstacles, such as social inequality, fragmented health systems, and limited resources. These challenges are reflected in the slow integration of liquid biopsy into clinical practice in the region, hampered by insufficient digital infrastructure and a strong reliance on public funding for translational research. Moreover, complex regulatory processes and lengthy clinical trial approval timelines hinder the timely implementation of innovations, further exacerbating inequities in access to advanced diagnostics and therapies (Werutsky et al., 2021; World Health Organization, 2020). As a result, many cases of cancer patients are diagnosed at later stages, leading to poorer outcomes and underscoring the urgent need for public policies that ensure equitable access to emerging diagnostic tools.

In conclusion, liquid biopsy offers a powerful tool for assessing minimal residual disease and monitoring treatment response, allowing for personalized real-time adjustments to therapeutic strategies. For patients with hard-to-reach tumors or those in compromised health, it provides a safer, less invasive alternative to traditional tissue biopsies, accelerating diagnosis and potentially improving clinical outcomes. As a result, liquid biopsy is becoming a key part of personalized medicine, especially on precision oncology, enabling the selection of targeted therapies based on each patient’s unique molecular profile, and potentially revolutionizing cancer diagnosis and treatment.
